# Cochlear shape reveals that the human organ of hearing is sex-typed from birth

**DOI:** 10.1038/s41598-019-47433-9

**Published:** 2019-07-26

**Authors:** J. Braga, C. Samir, L. Risser, J. Dumoncel, D. Descouens, J. F. Thackeray, P. Balaresque, A. Oettlé, J.-M. Loubes, A. Fradi

**Affiliations:** 1 AMIS, UMR 5288 CNRS-Université de Toulouse (Paul Sabatier), 37 Allées Jules Guesde, 31000 Toulouse, France; 2 Evolutionary Studies Institute, University of the Witwatersrand, PO WITS, Johannesburg, 2050 South Africa; 3 LIMOS, UMR 6158 CNRS-Université Clermont Auvergne, 63173 Aubière, France; 4 Statistics and Probabilities Team, Institute of Mathematics of Toulouse, UMR 5219 CNRS-Université de Toulouse (Paul Sabatier), 31062 Toulouse, France; 5 Clinique Pasteur, 45 Avenue de Lombez, 31076 Toulouse, France; 6 Department of Anatomy and Histology, School of Medicine, Sefako Makgatho Health Sciences University, Ga-Rankuwa, Pretoria, South Africa

**Keywords:** Evolutionary ecology, Biological anthropology

## Abstract

Sex differences in behavioral and neural characteristics can be caused by cultural influences but also by sex-based differences in neurophysiological and sensorimotor features. Since signal-response systems influence decision-making, cooperative and collaborative behaviors, the anatomical or physiological bases for any sex-based difference in sensory mechanisms are important to explore. Here, we use uniform scaling and nonparametric representations of the human cochlea, the main organ of hearing that imprints its adult-like morphology within the petrosal bone from birth. We observe a sex-differentiated torsion along the 3D cochlear curve in samples of 94 adults and 22 juvenile skeletons from cross-cultural contexts. The cochlear sexual dimorphism measured in our study allows sex assessment from the human skeleton with a mean accuracy ranging from 0.91 to 0.93 throughout life. We conclude that the human cochlea is sex-typed from an early post-natal age. This, for the first time, allows nondestructive sex determination of juveniles’ skeletal remains in which the biomolecules are too degraded for study but in which the petrosal is preserved, one of the most common bone within archaeological assemblages. Our observed sex-typed cochlear shape from birth is likely associated with complex evolutionary processes in modern humans for reasons not yet fully understood.

## Introduction

Signaling within a species influences decision-making, maximizes cooperative (e.g., sharing, communication) and collaborative (e.g., coordination) behaviors involved in sexual or other forms of social selection^1,2^. Therefore, complex evolutionary processes likely fine-tuned sex-differentiated neurophysiological and sensorimotor features involved in signaling among humans^2–6^. For instance, the human voice and hearing sensitivity are known as secondary sexual features^7–10^ but with yet unknown possible translations into the morphology of the main organ of hearing, the cochlea. The anatomical basis for sex-based differences in human hearing are therefore important to explore for any biologist and behaviorist interested to track its evolution in the fossil record and, in particular, the origin of the human uniquely “receiver-centered” hearing system that tailors speech content to needs of listeners^11,12^. Here, we investigated the shape in three dimensions (3D) of the human cochlea housed in a spiral-shaped cavity within the petrosal part of the temporal. Indeed, from birth, the cochlea is the organ that makes a more crucial contribution than the middle ear in setting the hearing range^13^. The cochlea also imprints details of its overall adult-like structure within the petrosal, the hardest and most dense bone in the mammal body that is most often preserved in fossil assemblages. Since the inner ear is not notably influenced by postnatal growth and development^14,15^, both juveniles and adults can be sampled and compared directly. We used a total of 94 X-ray medical computed-tomographies (CTs) representing adult individuals of African and European descent sampled in France and South Africa. Additional cochlear data were also taken from micro-CTs of 22 dry skulls representing juveniles ranging in age from birth to 10 years (Materials and Methods and SI 1).

Previous studies investigated human sexual dimorphism in cochlear size and gave contradictory results^16–18^. This was likely caused by the fact that none of these studies^16–18^ attempted to measure the allometric effect of the human sex-differentiated body mass on cochlear features. Moreover, previous analyses of cochlear variations among human populations did not filter out the scale and rotational effects to investigate potential differences in pure shape, as defined in detail by Kendall (1984) in his “theory of shape”^19^. All previously proposed approaches to cochlear morphology used finite-dimensional spaces represented either by a vector of features^20,21^ or parametric functions (e.g., logarithmic, polynomials, b-spline) based on angles and lengths^22^, leading to strong restrictions when performing statistical studies. As a first step, in order to overcome the drawbacks of the previously proposed approaches of cochlear morphology, we used a uniform scaling, a nonlinear registration and a statistical framework with a nonparametric representation of the 3D cochlear curve. As a second step, we further tested the applicability of our results for sex determination by measuring the accuracy of a method based on our observed sex-differentiated cochlear shapes. Indeed, sex determination from the human juvenile skeleton is an absolute prerequisite in most forensic, bioarchaeological and palaeoanthropological contexts. In the absence of sufficient biomolecule (DNA or peptides) survival, especially when fossil specimens are too old or degraded, this task is cumbersome because of the low degree of prepubertal skeletal sexual dimorphism among humans^23^, a limiting factor for successful sex determination. In forensic medicine, a minimal and arbitrary value of 0.95 is usually expected^24^ for accuracy (i.e., correct sex assessment). While some morphological methods show a relatively high accuracy of sex determination on adult skeletons^25^, this is not the case for the many methods that have been proposed for application to juvenile skeletons, especially for females^26–28^. Although some slight sexual dimorphism has been observed from an early post-natal age among humans^29,30^, there is still an important uncertainty about the age at which the human skeleton (especially the cranium) is quantifiably dimorphic. It is therefore well established that the juvenile skeleton cannot be sexed with any degree of accuracy from morphological observations alone^31,32^ without sex-typing from DNA^33^ or from peptides^34^. Indeed, any method for sex determination from the juvenile skeleton should be not only based on the description of purported sex-differentiated skeletal features but should also provide repeatable accuracy.

## Results

### Sexual dimorphism of the 3D cochlear shape

**Figure 1:**
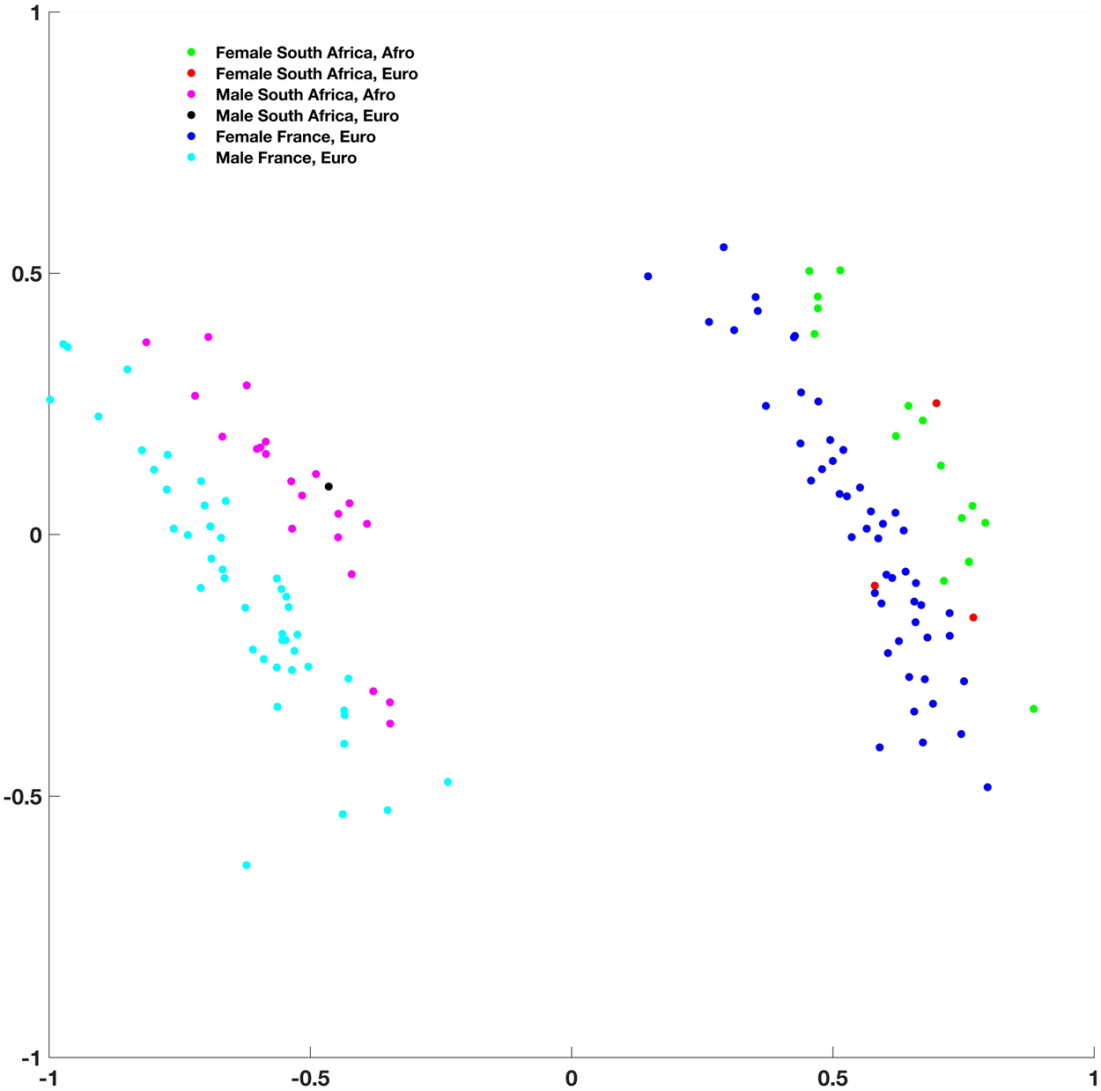
Unsupervised learning (clustering) (left) and tangent principal component analysis (TPCA) (right) of cochlear curves, demonstrating a clear distinction between males and females along PC1, irrespective of country of sampling (France or South Africa) or geographic origin (Euro: European, Afro: African).

We first used two samples of X-ray medical computed-tomographies (CT scans) representing adult individuals and collected in France (32 females and 22 males) and South Africa (15 and 3 females, 21 and 1 males of African and European descent respectively). We first tested the presence of sexual dimorphism in the 3D cochlear shapes in this sample of 94 adults by modeling mathematically the 3D cochlear shape by an open curve located along its outer periphery^20,35^. We then reduced the dimensionality of the 3D cochlear shape variables through unsupervised learning (clustering) and principal component analysis on the tangent space of shapes (TPCA, with the first and the second eigen projections) (Methods). We observed a clear separation between the curves representing adult males and females along the first Principal component (PC1) only, independently of the country of origin (France or South Africa) or the continent of origin (Europe or Africa) (Fig. 1 and Methods). When compared to PC1, the sixfold decrease of variation along the second Principal component (PC2) was caused by inter-individual differences within each sex and within each country of sampling. Therefore, since we eliminated differences in scaling to account for differences in body size between females and males but also between individuals within samples of females and males, sex was the primary source of 3D cochlear shape variation, more than any other parameter considered in our study.

### Visualization of sex-based differences in 3D cochlear shape

**Figure 2:**
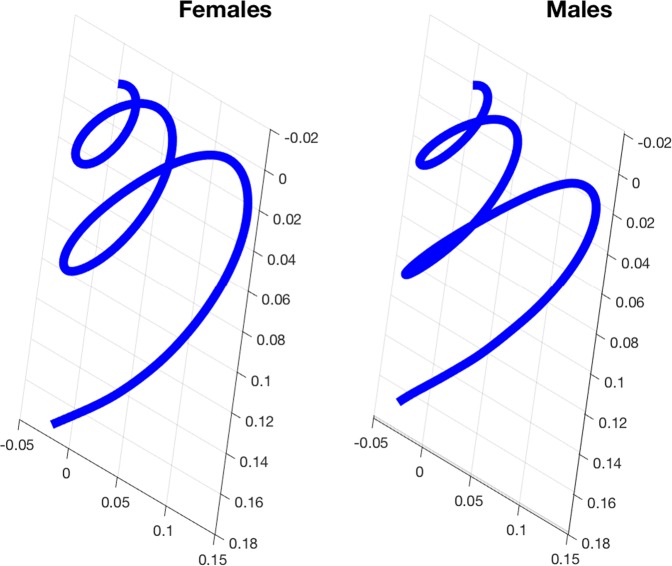
Fréchet means allowing us to visualize important shape differences between the female (left) and male (right) 3D cochlear curves shown here in exactly the same reference system and orientation.

**Figure 3:**
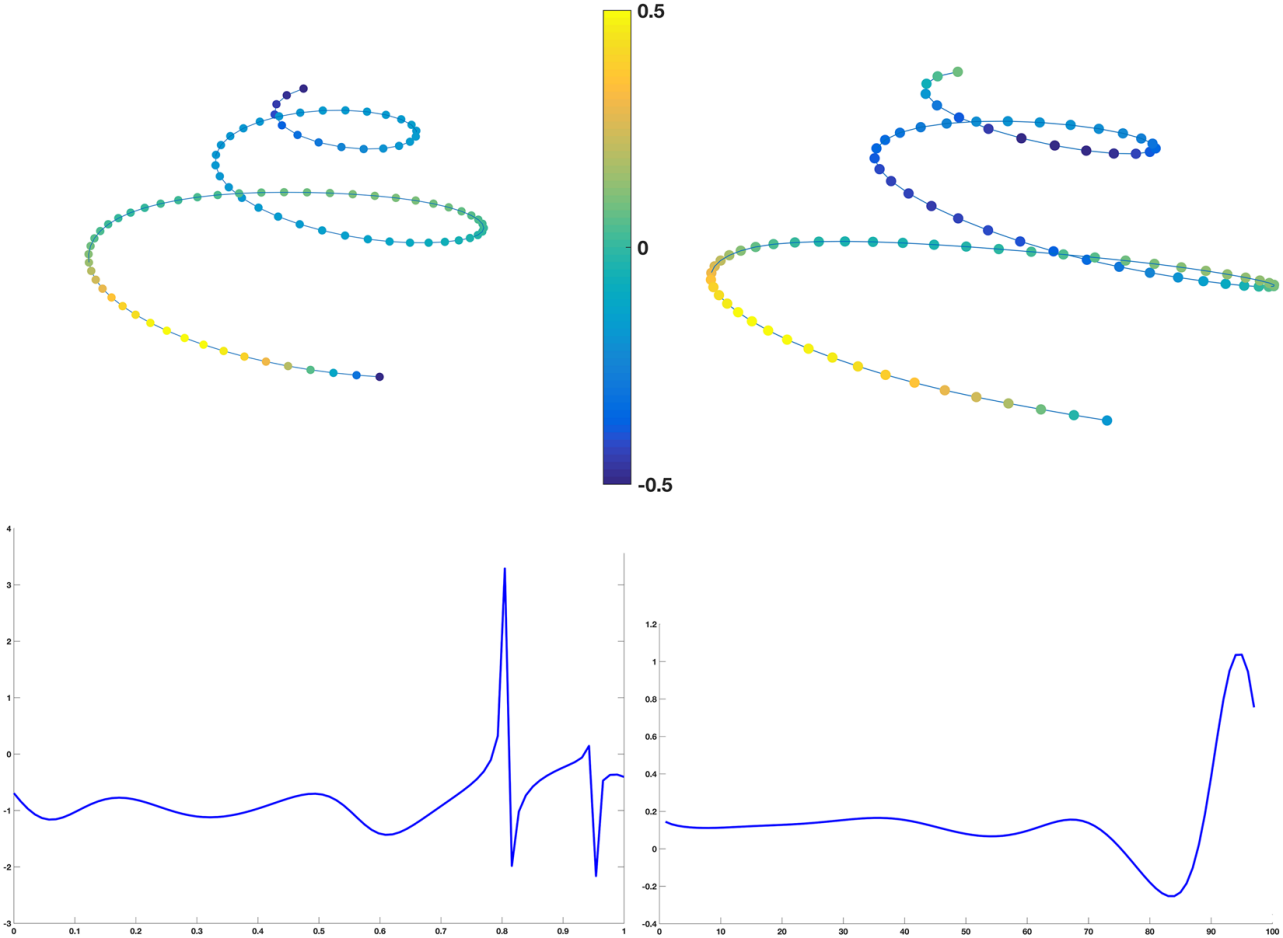
Fréchet means (top row) in females (left) and males (right) shown with a color-coded torsion function. Functions (bottom row) of the female to male index of torsion (left) and the torsion to curvature index (right) along the cochlear curve from basal (first decile) to apical (10^th^ decile) locations.

In a second step, in order to highlight local shape sex differences in torsion and curvature along the 3D cochlear curves that correspond to non-Euclidean spaces^36–38^, we computed the adult female and male Fréchet means for the observed cochlear curves sampled in this study (Fig. 2). Therefore, in contrast to previous studies^20–22^, we used a uniform scaling and a nonparametric representation of the 3D cochlear curve^35^. We used the Fréchet means as the underlying shapes^36^. This approach is both robust and accurate since the mean is an intrinsically unbiased representative. We observed that the sex-based differences in 3D cochlear shape were mainly due to differences in torsion (both bending and stretching) along the Fréchet means (Fig. 3). As shown from basal (first decile) to apical (10^th^ decile) locations along the normalized cochlear spiral curve, the most significant differences in torsion between the female and male Fréchet means were located between the 8^th^ and 10^th^ deciles and the highest sexual dimorphism appeared between the 8^th^ and 9^th^ deciles (Fig. 3). Moreover, the torsion to curvature index varied from location to location along the 3D curve and was highly variable between the 8^th^ and 10^th^ deciles (Fig. 3). This implies that 3D cochlear curves in humans do not follow a logarithmic function but rather a non-parametric one.

### Accuracy of our method of sex determination in adults

In a third step, for subsequent shape classification purposes, we measured the accuracy of our method for sex determination from the cochlear 3D shape. We therefore used 20 bootstrap trials of 20 adult females and 20 adult males (regardless of their geographic origin) for training while the remaining individuals (n = 54) were retained for sex assessment. In each trial, we computed the female and male Fréchet means. We learned the regression model from the training sample. We then applied it for sex assessment in the test sample. Overall, we obtained an accuracy of 0.93. When we considered the 20 trials of randomly selected text samples, the reliability ranged from 1 to 0.96 (with a mean value of 0.99) for males, and from 1 to 0.66 (with a mean value of 0.87) for females.

### Accuracy of our method of sex determination in juvenile skeletons

Finally, in a fourth step, in order to further test our method and determine its applicability to juveniles, we carried out the sex determination of a new sample comprising 22 juvenile skeletons of known sex (10 females and 12 males) ranging in age from birth to 10 years (Table 1). To do so, we used the female and male Fréchet means computed from our sample of 50 adult females and 44 adult males respectively, and we applied the regression model for the sex assessment of the 22 juvenile individuals. All the juvenile females were correctly classified and only two juvenile males were incorrectly classified as females (Table 1). Therefore, we obtained an accuracy of 0.91 for sex determination from the juvenile skeleton. It is important to note here that sex determination was correct for the few neonates in our sample (Table 1). Incorrect sex determination was not observed in a particular age category. Moreover, we obtained lower probabilities of individual sex determination for juvenile females (mean value of 0.20) than for juvenile males (mean value of 0.77 for the 10 correctly classified individuals) (Table 1).

**Table 1:** Reliability of our method of sex determination in 22 juvenile skeletons.

Individual	Known sex	Civil age	Sex determination	Probability of individual sex determination
Embr 583	Female	2 months	Female	0.253
Embr 308	Female	2 months, 15 days	Female	0.151
Embr 281	Female	1 year, 10 months	Female	0.326
Embr 384	Female	2 years, 1 month	Female	0.112
Embr 385	Female	5 months	Female	0.011
Embr 513	Female	7 months	Female	0.175
Embr 576	Female	7 months	Female	0.13
Embr 382	Female	9 months	Female	0.116
Embr 121	Female	5 years	Female	0.483
Embr 212	Female	5 years	Female	0.268
Embr 168	Male	Neonate	Male	0.702
Embr 249	Male	Neonate	Male	0.584
Embr 323	Male	Neonate	Male	0.678
Embr 277	Male	6 months	Male	0.587
Embr 388	Male	12 months	Female	0.384
Embr 479	Male	1 year, 3 months	Male	0.70
Embr 215	Male	7 months	Male	0.93
Embr 205	Male	3 years	Male	0.999
Embr 473	Male	3 years, 4 months	Male	0.67
Embr 383	Male	5 years, 4 months	Female	0.453
Embr 136	Male	6 years	Male	0.891
Embr 179	Male	10 years	Male	0.992

## Discussion

Our approach investigates for the first time 3D cochlear curves directly by their real (i.e., nonlinear and after a uniform scaling) shapes with two important advantages over previous methods^20–22^. First, our nonlinear elastic matching between shapes highlights local correspondences and differences by using full paths of deformations^35,38^. Second, our method allows the computation of optimal deformations in a 3D cochlear shape space. Optimal deformations represent the best correspondences between points across the two shapes as well as the mapping (vector field) that optimizes the deformation (transformation from one to another). We also visualize the shape means and covariances enabling a full statistical analysis. We observe that sex is the most dominant source of variation in 3D cochlear shape even when we consider separately each adult sample subset (e.g., African versus European descent, and France versus South Africa) (Fig. 1). Therefore, even though our sampling strategy was modest (i.e., sometimes with less than 30 males and/or females for a given geographic origin), here we provide the first statistical evidence for human sex-based differences in 3D cochlear shapes observed in multilingual and cross-cultural contexts (Materials). In this context and in the absence of reliable and accurate enough morphological methods of sexing from the human juvenile skeleton, the results presented here are important. If confirmed by more modern human samples from other geographical origins and in other species of great apes, our results could open new perspectives into the assignment of biological sex to important fossil individual specimens attributed to our own genus *Homo* or to other genera (e.g., *Australopithecus*, *Paranthropus*) and for which sex-typing from DNA or from peptides is not (yet) possible. In contrast, the possible absence of sex-typed cochlear shape in other species of great apes would suggest that social structure, mating systems and communication in these species did not affect the morphology of this organ during evolution. Moreover, in the absence of pre-existing knowledge (i.e., supervised learning using training samples representing modern specimens of known sex), yet unknown patterns of variation in cochlear shapes within fossil hominin species could be still investigated. In this case, unsupervised learning techniques could use cochlear shapes directly measured in fossil hominin samples for sex assessment of particular specimens.

The ontogeny of the human skeletal sexual dimorphism in size, structure and shape is still the subject of ongoing debate^29,30^. Distinct parts of the human skeleton, especially the cranium, not only display differential degrees of sexual dimorphism at a given stage of growth and development, but also independent growth patterns in size and shape that may also vary with sex^39^. Little is known about the potential sexual dimorphism of the morphology of the human hearing system. Therefore, we focused our attention on the cochlea because it represents the only sensory system organ that imprints details of its overall adult-like structure within bone by term birth^14,15^. It is better amenable to evolutionary studies of communication systems for three main reasons. First, this organ acts as a frequency analyzer of incoming sounds into high and low frequencies along its spiral canal from basal to apical locations respectively^40^. Second, extensive studies in placental mammals (including primates) demonstrate the possibility to estimate hearing capacities for gross dimensions and shape of the cochlear cavity within bone^40,41^. Finally, the cochlear overall structure is also accessible in juvenile and adult fossil hominins^20,42^. Given the sexual differences in the human fundamental and resonance frequencies^7,8^, the unique human sexual dimorphism caused by a male disproportionate glottal-size^43^ (with 50–60% difference between adult females and males, a value clearly out of proportion to the average human sexual dimorphism in height or other linear dimensions), it was important to investigate whether these secondary sexual features translated into the morphology of the cochlea. Little emphasis has been laid on human sexually selected communication systems, their relationships with signal reliability (“honesty”) and sociocultural behaviors, particularly on how women and men perceive others in gendered societies through signals emitted to represent the sender’s characteristics^44–46^. In non-human primates, male vocal signals mediate mate choice^47,48^ and voice pitch is perceived as an indicator of mate quality^45,49^ while vocal attractiveness is correlated with both body and facial attractiveness in humans^50,51^. In multilingual and cross-cultural contexts, women find men with a more masculine voice more attractive^45,46,49^. Our results may suggest that sexual selection has played an as yet unrealized role in the evolution of a sex-differentiated human hearing system from birth.

The differences highlighted in our study may induce sex-based hearing properties. However, cochlear mechanics in humans are still debated^52^ and structure-function relationships within the cochlea should be considered with caution. When compared to other extant and extinct apes, humans have a longer cochlea than expected for their body mass, a proxy indicating a major auditory change that likely occurred in early representatives of the genus *Homo* approximately 2 million years ago^20^. This finding is consistent with the unique human sensitivity at low-frequencies^41,53^. If we posit a sender-receiver co-evolution, the hearing organ may reveal important evolutionary trends in the human sound communication system, including speech. Should our proposed evidence for a sex-differentiated cochlea among humans be associated with neurobiological and social underpinnings of our unique sexual dimorphism in the production and the reception of sounds, it would facilitate the understanding of key features of human communication systems.

## Materials and Methods

### Experimental design and sampling

Our sampling strategy aimed to investigate potential differences in 3D cochlear shapes caused by (i) sex, (ii) country of sampling (France or South Africa) and (iii) continent of origin (African and European descent) for the sample from South Africa only. For both samples (France and South Africa), all the patients were adults (i.e., older than 18 years of age) and their identity was made anonymous to remain strictly confidential. Given the legal frameworks relating to medical data protection issues in France and in South Africa, no information about “ethnicity” was available for either samples. “Ethnicity” is a self-ascribed category by which individuals seek to assert their identity. It is here considered as highly imprecise, inconsistent and defined differently in different places and at different times. However, in South Africa, the public and private registrations require South Africans to indicate their “race” (a concept with no validity in human biology) by ticking one of four boxes (“Whites”, “Blacks”, “Coloureds” and “Asians”). Since this information was available, we could consider the “continent of origin” (either Europe for “Whites” or Africa for “Blacks”) as a third additional stratification variable (in addition to sex and country of sampling) for the sample from South Africa only (“Coloureds” and “Asians” were underrepresented at the hospital concerned and were not included). We nevertheless considered this information with caution since it was self-ascribed by the patient. Moreover, the language(s) spoken by the patients were not known because the medical data were collected retrospectively. However, the sample from South Africa was collected from the Steve Biko Academic Hospital in Pretoria by one of us (AO), one of its faculty members at the time of sampling. This hospital is a public health and training institution that provides tertiary healthcare to the multicultural population living in Pretoria. It is therefore representative of the pattern of diverse linguistic groupings in this city. Indeed, the official languages in South Africa include English and Afrikaans (derived from Dutch) and nine Southern Bantu languages; Zulu being the most spoken as a first language, followed by Xhosa. Afrikaans and English are respectively the third and fourth most common first language in South Africa. South Africa, children usually learn in their mother-tongue at school for the first three years (a Southern Bantu language more than 60% of children). They then switch to either English or Afrikaans and continue with that language for the rest of their schooling career.

### Ethical approval

All the steps of the present study were performed in accordance with relevant guidelines and regulations. No data used in this study involved experimentation, intervention, risk or constraint added by the research. For both samples (France and South Africa), the information regarding sex and continent of origin was tabulated and used for statistical purposes only. Our research was non-interventional and was based on anonymous medical data collected retrospectively. In France, the processing of personal data in the healthcare sector for research must be authorized by the National Commission of Informatics and Civil Liberties (CNIL). Moreover, since our data were only digital, anonymized and collected retrospectively, the approval of the committee for the protection of persons (a French ethics committee) was not necessary. In order to simplify the authorization procedure, the CNIL has adopted the reference methodology MR-003 for data collections of healthrelated data for non-interventional research purposes (https://www.cnil.fr/sites/default/files/atoms/files/mr-003.pdf). For this study, we complied with the reference methodology MR-003. In South Africa, we obtained ethical clearance in 2017 from the Research Ethics committee of the Faculty of Health Sciences, University of Pretoria. Ethics Reference No: 465/2017.

The adult sample is represented by CT scans collected retrospectively in the Clinique Pasteur, Clinique Saint Jean du Languedoc and Médipôle (Toulouse, France), and the Steve Biko Academic Hospital (Pretoria, South Africa). For the sample from France, the CT scans were performed between 2012 and 2014, mainly to visualize the maxillary sinuses. For the sample from South Africa, the CT scans were performed in 2016. The pixel size ranged from 0.46 to 0.52 mm with a maximum slice thickness of 2 mm.

The juvenile sample is represented by micro-ct scans of crania curated in the « *Institut d’Anatomie Normale et Pathologique* » of the University of Strasbourg. These skeletons correspond to individuals of known sex and age at death (catalogued with the « Embr » prefix and listed in Table 1). This sample was collected mainly by Professors HWG Waldeyer (1836**–**1921) and G Schwalbe (1844**–**1916) before 1918^54^ (Rampont 1994). We obtained permissions to access these skeletons that have already been used in several published studies^16^. The micro-ct scans (pixel size of 0.041 mm) were obtained from the XtremeCT (Scanco Medical; http://www.scanco.ch) system at the Institut de Médecine et de Physiologie Spatiales, Toulouse, France (http://www.medes.fr/).

### Data processing

We first imported the CT scans into the Avizo software package (www.vsg3d.com/avizo) for the segmentation and the 3D reconstruction of the air-filled cochlea. We decided arbitrarily to only use the left cochlea and we assumed that sexual dimorphism also occurred on the right side. We used a threshold of 550–650 Hounsfield Units to extract the bone structures and for further reconstructions. The cochlear size and shape were modelled following a method previously used in Braga *et al*.^20^ by a series of landmarks placed at small intervals along the outer periphery of the cochlea, between the apex and the point marking the origin of the basal turn (where there is a saddle between the cochlear part and the vestibule) very close to the inferior margin of the round window. We subsequently analyzed the cochlear 3D shape by imposing appropriate geometric invariances including rigid motions, uniform scaling, and re-parametrization. Here, we point out that there is a difference between methods that perform registration based on features vectors and those that perform intrinsic statistical shape analysis. For both registration methods, the main goal is to establish correspondences between pairs of observations. Comparisons in a Euclidean space for methods based on features vectors lead to statistical analyses where the template is either selected as a predetermined observation from the sample, or constructed using Euclidean averages. In this case, a major drawback consists in ignoring the intrinsic complexity and nonlinearity caused by the structure of the data. The aim of the intrinsic shape analysis conducted in our study was to (i) achieve accurate registration with uniform scaling and correspondences between shapes, (ii) focus on the representation of the shape, (iii) analyze shape on a nonlinear manifold. Thus, the objectives of such intrinsic shape analysis encompassed correspondence-based registration methods while simultaneously integrating the tools for statistical analysis into a nonlinear registration framework.

We further investigated the robustness of our proposed method when used to assess sex accurately (i.e., to classify unknown trials) from the 94 adult cochlear curves sampled in this study. We evaluated the classification performance depending on the average estimates. We repeated these experiments 20 times to avoid a biased sampling (and we reported the mean and the standard deviation of these repeated experiments). In the Supplementary Figure (Fig. S1), we give one example out of the 20 trials and we show the misclassified 3D cochlear curves. For this trial, we observed three misclassified shapes for women and only one for men. The likelihood ratio values were close to the threshold 0.5. This implied a poor precision for these observations. We can report more favorably that the model achieved high accuracy for the rest of the test sample.

### Statistical analysis

The dimensionality reduction of the population shape variables through tangent principal component analysis (TPCA) allowed local geometric shape analysis to characterize infinitesimal changes along the cochlear curve, but also highlighted global changes in shape covariates using the dominant modes of variation. The key advantage of this approach was that it allowed the use of 3D cochlear shape as a geometric phenotype in more sophisticated multivariate analyses. While the choice of TPCA for representing the tangent space was made for computational convenience, it is important to note that PCA yields a basis that maximizes the feature variance in the sample along each projection. The study of shape variance shows discriminatory rules that help in classifying groups. Additionally, even if the 3D shape analysis was performed locally on the curves, the principal projections captured global shape properties in the data, and hence helped to interpret appropriately the variability in 3D cochlear shapes. The application of our analysis to samples of 3D shapes demonstrated both global and local effects of sex for 3D cochlear shape variation.

The Fréchet mean (also called Karcher mean)^35–37^ was then used to capture the characteristic of the 3D cochlear curves for a given sample. It was also used as a common reference template for registering the individual shapes to perform a group analysis. Instead of being a subject-specific template, the Fréchet mean represents a statistical template that minimizes the shape variance. The main advantage of this approach is that it also allowed us to explore the covariance structure of the 3D cochlear shapes in an intrinsic manner. All the computations were made using programs written in C/C++ and Matlab 2016a. We used a regression model on the shape space. It is known that these models depend on unknown re-parametrization functions (or domain deformations)^55^ which remain a challenging task. For instance, in a regression case where the shape is available as a finite number of observed data, we are interested in studying their re-parametrizations for describing the underlying shape of any newly observed input. For shape regression, each of the observed shapes comes with its own reparametrization. We were interested in computing the Fréchet mean of re-parametrization and in learning a Gaussian process on the space of cumulative distributions as a subspace of the unit sphere. Furthermore, we used a Bayesian inference for tuning the regression model, one among the most effective existing solutions particularly for regression and classification of shapes.

## Supplementary information

Example of misclassified males and females during one trial.

## Data Availability

The datasets generated during and/or analyzed during the current study are available from the corresponding author on reasonable request.
