# Supplementary material for: Cochlear shape reveals that the human organ of hearing is sex-typed from birth
Source: Sci Rep. 2019 Jul 26;9:10889. doi: 10.1038/s41598-019-47433-9 (PMC6659711; doi:10.1038/s41598-019-47433-9)

Title

Cochlear shape reveals that the human organ of hearing is sex-typed from birth.

Authors

J. Braga[1,2]*†, C. Samir[3]†, L. Risser[4], J. Dumoncel[1], D. Descouens[5], J.F. Thackeray[2], P. Balaresque[1], A. Oettle[6], J.-M. Loubes[4], A. Fradi[3]

Affiliations

1. AMIS, UMR 5288 CNRS-Université de Toulouse (Paul Sabatier), 37 Allées Jules Guesde, 31000 Toulouse, France. jose.braga@univ-tlse3.fr, jean.dumoncel@univ-tlse3.fr, patricia.balaresque@univ-tlse3.fr

2. Evolutionary Studies Institute, University of the Witwatersrand, PO WITS, Johannesburg, 2050, South Africa. mrsples@global.co.za

3. LIMOS, UMR 6158 CNRS-Université Clermont Auvergne, 63173 Aubière, France. chafik.samir@udamail.fr, anis.fradi@etu.uca.fr

4. Statistics and Probabilities Team, Institute of Mathematics of Toulouse, UMR 5219 CNRS-Université de Toulouse (Paul Sabatier), 31062 Toulouse, France. loubes@math.univ-toulouse.fr, laurent.risser@math.univ-toulouse.fr

5. Clinique Pasteur, 45 Avenue de Lombez, 31076 Toulouse, France. archaeodontosaurus@live.fr

6. Department of Anatomy and Histology, School of Medicine, Sefako Makgatho Health Sciences University, Ga-Rankuwa, Pretoria, South Africa. anna.oettle@smu.ac.za

Corresponding author: José Braga, jose.braga@univ-tlse3.fr

† These authors contributed equally to this work

Supplementary Figure S1

Example of one trial (out of 20 ones) which revealed only one misclassified male (1 curve out of 20) (right) but three misclassified females (3 curves out of 20) (left).

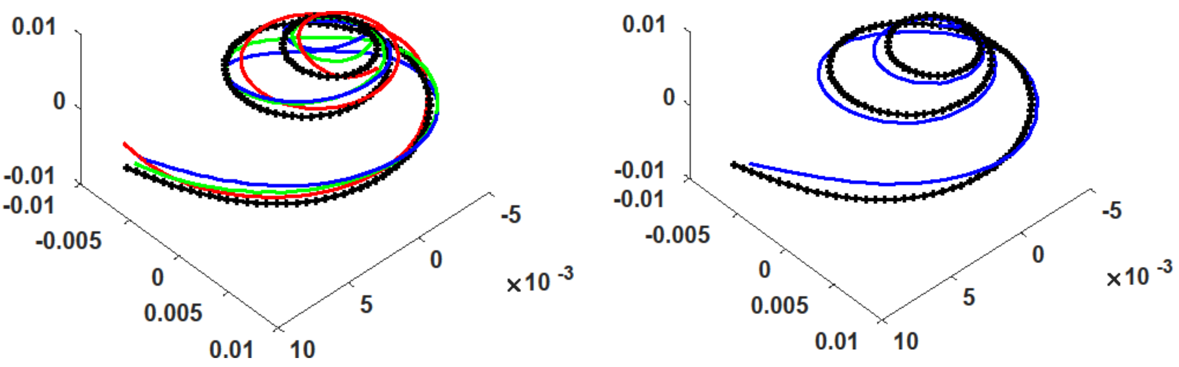

Supplement: Supplementary file 1 — Example of misclassified males and females during one trial. [file 41598_2019_47433_MOESM1_ESM.pdf]
